# Prediction of range expansion and estimation of dispersal routes of water deer (*Hydropotes inermis*) in the transboundary region between China, the Russian Far East and the Korean Peninsula

**DOI:** 10.1371/journal.pone.0264660

**Published:** 2022-04-14

**Authors:** Ying Li, Yuxi Peng, Hailong Li, Weihong Zhu, Yury Darman, Dong Kun Lee, Tianming Wang, Gleb Sedash, Puneet Pandey, Amaël Borzée, Hang Lee, Yongwon Mo

**Affiliations:** 1 Tiger and Leopard Conservation Fund in Korea (KTLCF), and Research Institute for Veterinary Science, College of Veterinary Medicine, Seoul National University, Seoul, Republic of Korea; 2 College of Geography and Ocean Science, Yanbian University, Hunchun, Jilin, China; 3 National Forestry and Grassland Administration Key Laboratory for Conservation Ecology in the Northeast Tiger and Leopard National Park, Beijing, China; 4 Amur Branch of World-Wide Fund for Nature, Vladivostok, Russia; 5 Department of Landscape Architecture and Rural System Engineering, Seoul National University, Seoul, Republic of Korea; 6 College of Life Sciences, Beijing Normal University, Beijing, China; 7 Northeast Tiger and Leopard Biodiversity National Observation and Research Station, Beijing, China; 8 Land of the Leopard National Park, Vladivostok, Russia; 9 Laboratory of Animal Behaviour and Conservation, College of Biology and the Environment, Nanjing Forestry University, Nanjing, People’s Republic of China; 10 Department of Forest Resources and Landscape Architecture, College of Life and Applied Sciences, Yeungnam University, Gyeongsan, Republic of Korea; Amity University, INDIA

## Abstract

Global changes may direct species expansion away from their current range. When such an expansion occurs, and the species colonizes a new region, it is important to monitor the habitat used by the species and utilize the information to updated management strategies. Water deer (*Hydropotes inermis*) is listed as Vulnerable species in IUCN Red List and is restricted to east central China and the Korean Peninsula. Since 2017, water deer has expanded its range towards northeast China and the Russian Far East. The objective of our study is to provide support for a better understanding of habitat use and provide suggestions for developing conservation strategy. We collected occurrence data in northeast China and the Russian Far East during 2017–2021. We used MaxEnt to predict habitat suitability for water deer and applied Circuitscape to determine possible dispersal routes for the species. We used seven environmental variables, viz., altitude, slope, aspect, distance to built-up area, distance to water source, distance to cropland and distance to roads for habitat suitability prediction. We chose the MaxEnt model (AICc = 2572.86) suitable for our data with the AUC value result of 0.935±0.014. There is good quality habitat for water deer in the boundary area of the Yalu and Tumen River estuaries between China, North Korea, and the Russian Far East, as well as the east and west regions of the Korean Peninsula. We identified three main suitable habitat patches, two of them located in east (NK2) and west (NK3) North Korea, and one in the newly colonized area downstream of the Tumen River along the border of China, Russia, and North Korea (TM1). Elevation, distance to cropland and water sources, and presence of wetlands were the variables that positively contributed to modelling the suitable habitats. Two possible dispersal routes were determined using the circuit theory, one was across the area from North Korea to the downstream Tumen transboundary region (Route B), and the other was across North Korea to the boundary region in China and along the tiger national park in northern China (Route A). A series of protected areas in North Korea, China, and Russia may support the dispersal of water deer. From the study on water deer dispersal, we can understand the existing ecological network in northeast Asia, which will benefit the whole landscape and biodiversity conservation. However, there are many threats present, and there is need for continued monitoring inside and outside the protected areas. Information sharing with stakeholders and carrying out local communities awareness activities are important. The establishment of a Northeast Asia landscape conservation network would help establish monitoring and conservation planning at a broad scale, and this study provides an example of the need for such a network.

## Introduction

Herbivorous animals play an important role in the ecosystem in terms of direct ecological interaction with animal predator and vegetation used for food. Herbivores also have indirect interactions with other species, such as insect biodiversity [1, 2]. Monitoring the habitat used by herbivores is necessary for conservation of the entire ecosystem [3]. Human activity, climate change and population growth of the focal species may cause species to shift from their original distribution area [4, 5]. An important reason for monitoring is that when a species first occupies a new region, it will exhibit clearer habitat preferences compared to areas when they are in areas where it has long been established.

Water deer (*Hydropotes inermis*) is a species endemic to East China (Chinese water deer, *H*. *i*. *inermis*) and the Korean Peninsula (Korean water deer; *H*. *i*. *argyropus*). The water deer is listed as ‘Vulnerable’ at a global level by the IUCN Red List [6]. The population of Chinese water deer is decreasing and occurs in a fragmented habitat [7]. The main distribution area of the Korean water deer is the Korean Peninsula [8]. In South Korea, water deer are known to be a widespread species, as they occupy most of the habitats, including wetlands, grasslands, and forests [9]. In North Korea, water deer is listed as wildlife with economic value and has been provided governmental support for protection in the wild [10]. Water deer populations were historically distributed in western North Korea, and this species has translocated three times to the east of North Korea in 1968 [10]. In 2005, the presence of water deer was reported from protected areas located in eastern North Korea, including Cheonbul Mountain Animal Reserve in South Hamgyeong Province and Daegak Mountain Animal Reserve in North Hwanghae Province [11]. There are no specific occurrence data or population descriptions of water deer in North Korea. However, on the basis of available references, it can be assumed that water deer is distributed in suitable habitats in both western and eastern North Korea [6, 11]. However, since 2019, water deer has been frequently reported in the boundary area between China, Russia, and North Korea, areas where there are no earlier formal records of this species [12]. In the newly occupied areas, in the lower Tumen River basin in China and in the Russian Far East, there are still many wild areas with relatively low human impact [13]. As herbivores, water deer contributes to ecosystem functions, and they can be a potential prey for big cats such as tigers and leopards in the national parks in the boundary area between China and Russia [12, 14, 15]. Study of the habitat use can provide information on the potential expansion trend and dispersal routes [16–18]. Understanding the habitat of this species is essential for management and conservation of local wildlife and ecosystems [19]. Water deer prefers wetlands, swamps, low lands, and grasslands, and also occupy farmlands [20]. Along the Yalu and Tumen rivers, new water deer sighting are being records, which may due to large areas of suitable habitat along the riverbanks. As this deer species can quickly increase in population size [21], it may become an important mid-sized mammal species in newly occupied territories, and affect river and wetland ecosystems, and may also impact the distribution of other wildlife.

There is an urgent need to understand the changes in habitat condition due to new expansions of population and predict the distribution of the expansions and the possible dispersal route. Species distribution model and landscape analysis can help provide the knowledge by considering the species and their environmental variables. Using the result of species distribution model, we can identify the important habitats for the species and also understand influential factors, which will be important for management strategies. Species distribution models can help determine conservation priorities in North Korea, as done for amphibians [22]. Species distribution modeling (SDM), also called environmental, bioclimatic, species niche, or habitat suitability modeling [23], and can be used to assess the habitat use of a species. The results can reveal the factors that may influence the species, and thus may provide important references for management of the species [24]. SDM can also be applied to predict species dispersal under climate change scenarios, as well as to model the results of changes in land use changes [25]. Climate change data and land use prediction data are often used separately, but numerous unexpected biases may be included because of limitations in the knowledge of species population size or land cover connectivity [26, 27]. Maximum entropy (MaxEnt) is a widely used machine learning method for SDMs, and it was specifically developed for presence-only data to overcome the problems of small undesigned samples [28].The core idea is to take full account of the known information when inferring the distribution of unknown probabilities and treating the unknown information indiscriminately [29]. Habitat suitability level can also be predicted, thus enabling the calculation of difference in habitat and resource, between protected and non-protected areas [30]. Circuit theory is a recently developed technique that can quantify movement across a landscape and can be applied in many fields, including landscape ecology, population genetics, movement, and fire behavior [31]. This method is based on random walk and graph theories [32]. A circuit is usually defined as a network of nodes connected by resistors and is used to analyze graphs.

We hypothesize that the adequate habitat for water deer in and around the current region of expansion contributes to the northward expansion of this species. Our research objective is to understand the factors facilitating the range expansion of water deer. It is imperative to understand the habitat preferences of the species and predict the range expansion in order to guide management strategies for monitoring and research in the newly occupied areas. We used a MaxEnt model to predict water deer habitat characteristics, and the circuit method to analyze the conservation area network in the Tumen transboundary region. The results provide an overall understanding of the potential habitat and possible dispersal routes of water deer, which can be used for further monitoring and conservation strategies for wildlife on a larger scale.

## Material and methods

### Research overview and data processing

**Fig 1:**
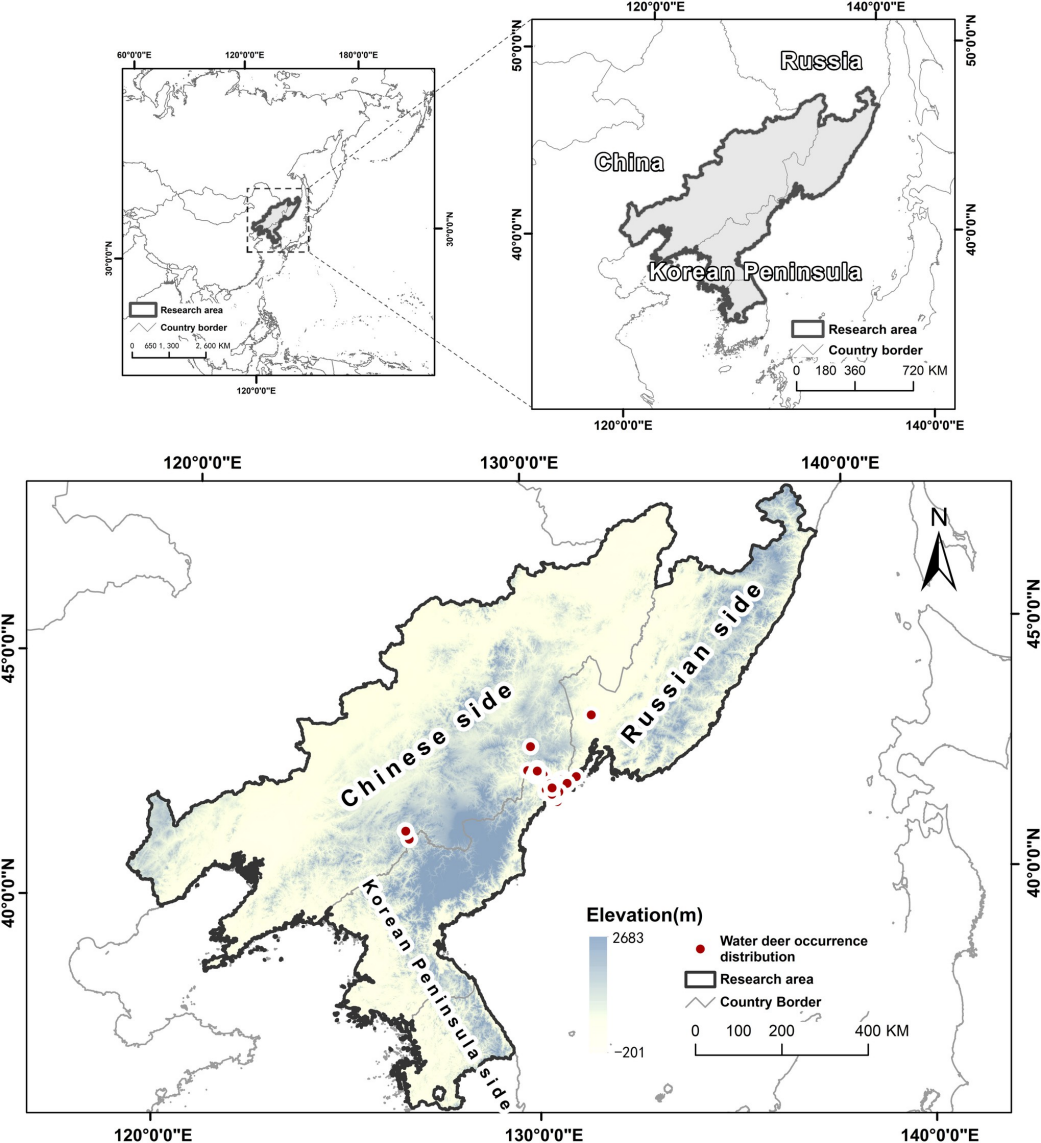
Locations of water deer occurrence used for modeling. Base layer was created based on SRTM elevation data in ArcMap 10.3 (desktop.arcgis.com; ESRI, Redlands, USA). Occurrence locations were from author’s data (S1 Table) and based on published data source [12, 14, 34] as well as GBIF database (DOI: 10.15468/39omei).

Our research area focused on the new expansion area including the southern regions of Liaoning, Jilin, and Heilongjiang provinces in China, the Primorye region in Russia, the whole of North Korea, and the northern part of South Korea (34.70317°N–49.008799°N, 117.65389°E–139.26630°E). To predict water deer habitat suitability, we first need geolocated occurrence data, and so we collected 102 occurrence data in total (Fig 1). The occurrence data was obtained from various sources, including ecological monitoring, camera trapping records, roadkill and hunting records, as well as published literature records [14, 33, 34] and the public database Global Biodiversity Information Facility (GBIF) [35]. To avoid overfitting when conducting the habitat model, we deleted datapoints which they were less than 500 meters away from each other. This threshold is based on the size of the home range of the water deer, around 1 km^2^ [36]. Subsequently, 75 data points were used for the analysis [37].

Considering the water deer ecology in southeast China and South Korea, seven main environmental variables (Table 1) were selected for habitat modelling, viz., altitude, slope, aspect, distance to built-up areas, distance to water source, distance to cropland and distance to roads [21, 38, 39]. The selection of environmental variables considered the ecological requirements of water deer and include natural and human influenced factors. The natural factors related to the topographical factors such as altitude, slope, aspect, and the needs for water. Human influenced factors may affect the habitat use by wildlife. We used distance to built-up areas, distance to roads and distance to cropland as the human influenced factors. The three topographic data utilized were altitude, slope, and aspect, which were processed from the SRTM 90-meter resolution World DEM database [40].

**Table 1:** Water deer environmental variable information.

Variable category	Variable name	Description	Data source
Landcover	Landcover	19 different land cover types	GLCLU (https://glad.umd.edu/dataset/global-land-cover-land-use-v1)
Topography	Altitude (DEM)	Elevation of site area(m)	SRTM (https://srtm.csi.cgiar.org/)
	Slope	Slope of site area (°)	
	Aspect	Aspect of the area (flat, north, east, west, south)	
Proximity	Distance to cropland	Distance from cropland (m)	GLCLU (https://glad.umd.edu/dataset/global-land-cover-land-use-v1)
	Distance to built-up	Distance from built-up areas (m)	
	Distance to water	Distance from rivers, streams, and lakes(m)	OpenStreetMap (https://www.openstreetmap.org/)

Altitude and slope variables were continuous data, and aspect was classified into five categories ranging from to 0–360°: 1- flat (−1–0.0001°), 2- north (315–360°), 3- east (45–135°), 4- south (135–225°), and 5- west (225–315°). Land cover data was processed from global land cover [41] and land use data of 2019 with 19 land cover types, viz., desert, semi-arid, dense short vegetation, open tree cover, dense tree cover, tree cover gain, tree cover loss, salt pan, wetland sparse vegetation, wetland dense short vegetation, wetland open tree cover, wetland dense cover, wetland tree cover gain, wetland tree cover loss, ice, water, cropland, built-up area, and ocean [41]. Distances from built-up areas, cropland, and water source were processed using the ArcGIS Euclidean distance toolkit in ArcGIS 10.3 (ESRI, Redland, CA, USA),with data from land cover and the open street map database as sources [41, 42]. To obtain a high-quality predictions, and since strongly correlated environmental variables can lead to model overfitting, we conducted a Spearman’s correlation test using the IBM SPSS software version 26.0 [43]. Selected seven uncorrelated environmental variables were selected to estimate habitat suitability (threshold = 0.7). The variables thus selected were aspect, slope, distance to water, distance to cropland, distance to built-up area, elevation, and landcover. Considering the home range of water deer, we selected a 500 m resolution for the analyses [36], and we used the 2000 Korean Central Belt 2010 projection system.

### Habitat suitability

In this study, we used MaxEnt 3.4.1 software [44] to build the model to predict habitat suitability. We uploaded the 75-occurrence points data and the seven uncorrelated environmental variables to the software, and we set the output format as Logistic, using 25 percent species occurrence data as test data set. MaxEnt performance is closely related to both the regularization multiplier parameter and feature selection [45]. The ENMeval R package was applied to test the best model parameters by setting it to select the model with the most significant result, omission value less than 5%, and delta AICc value less than 2 [46]. The best model setting was selected from 1160 model’s test. The parameter setting used the linear, quadratic, and threshold features together, with the regularization multiplier value set to 0.9. We built the model with 10,000 background points and 10 bootstrap replicates. A Jackknife analysis was used to assess the contribution of environmental variables. The model performance was evaluated using a threshold-independent area under the curve (AUC) of the receiver operating characteristic curve (ROC). The Jenks method was employed to determine habitat suitability level using ArcGIS 10.3 (ESRI, Redland, CA, USA).

### Habitat connectivity

Habitat connectivity was analyzed to predict suitable habitat connections, using the Appling landscape connectivity analysis method. Based on Ohm’s law, voltage V is applied across a resistor, current I flows through it, and the current intensity depends on the voltage V and resistance R [47]. In the application of circuit theory to landscape ecology, complex landscapes can be considered as the conductance, and the species randomly travelling to different directions can be considered as the random walker. Ecological features that are beneficial (positive) for the movement of the focal species, such as preferred land cover types, are given low resistance values, whereas negative features are given high resistance values.

Connectivity was analyzed for three area with high-quality habitat, as defined using MaxEnt results. They were new expanding Tumen River region (TM1), East North Korea region (NK2) and West North Korea region (NK3). The newly occupied areas in the lower Tumen region were defined as target region TM1. The predicted high-value habitat in the west and east coasts of the Korean Peninsula, for which there are also records of water deer [6, 11], were defined as regions NK2 and NK3 respectively [6, 11]. The three high quality habitat patches regions TM1, NK2 and NK3 were designated as nodes, and thus the target regions for the analyses on connectivity. The three patches were given specific values with value of 1, 2, and 3. The background values were set as 0 and the layer was saved as ASCII file.

For the background we had to define resistance surfaces to calculate the resistance between the nodes. The surface resistance can also be calculated from the MaxEnt modelling results [48], We used the surface values derived from the MaxEnt results using the formula R = 101 − M, where R is the surface resistance and M is the MaxEnt value*100. Because MaxEnt values multiplied by 100 ranged from 0 to 100, the resistance surface values ranged from 1 to 101 [49]. The calculations were conducted in ArcGIS Raster calculator. The resistance surface and node map were converted to the ASCII data and uploaded to the Circuitscape ArcGIS toolkit to determine the connection map [50], with all settings as default.

## Results

### Habitat suitability

The ROC curve of the model had an AUC value of 0.935±0.014, indicating that our model could accurately simulate the relationship between the geographical distribution of water deer and the factors analyzed. The three most important variables selected for the model were elevation and distances to cropland and water source (Table 2). Elevation had the highest percentage of contribution to the model (59.6%), followed by distance to cropland (16.3%), land cover (10.8%), and distance to water sources (8.8%). The contribution of the three variables, via., slope (2.1%), f aspect (1.6%) and distance to built-up areas (0.7) make up only 4.4% to the model. This indicate that these three variables were less important for the model compared to the other variables.

**Table 2:** Environmental variable contribution percent and permutation importance used for water deer habitat suitability modelling. Based on 99 distribution locations in Northeast China and the Russian Far East.

Variable	Percent contribution	Permutation importance
DEM	59.6	64.8
Distance to cropland	16.3	13.4
Landcover	10.8	5.9
Distance to water	8.8	10.4
Slope	2.1	3
Aspect	1.6	1.8
Distance to built-up	0.7	0.7

**Fig 2:**
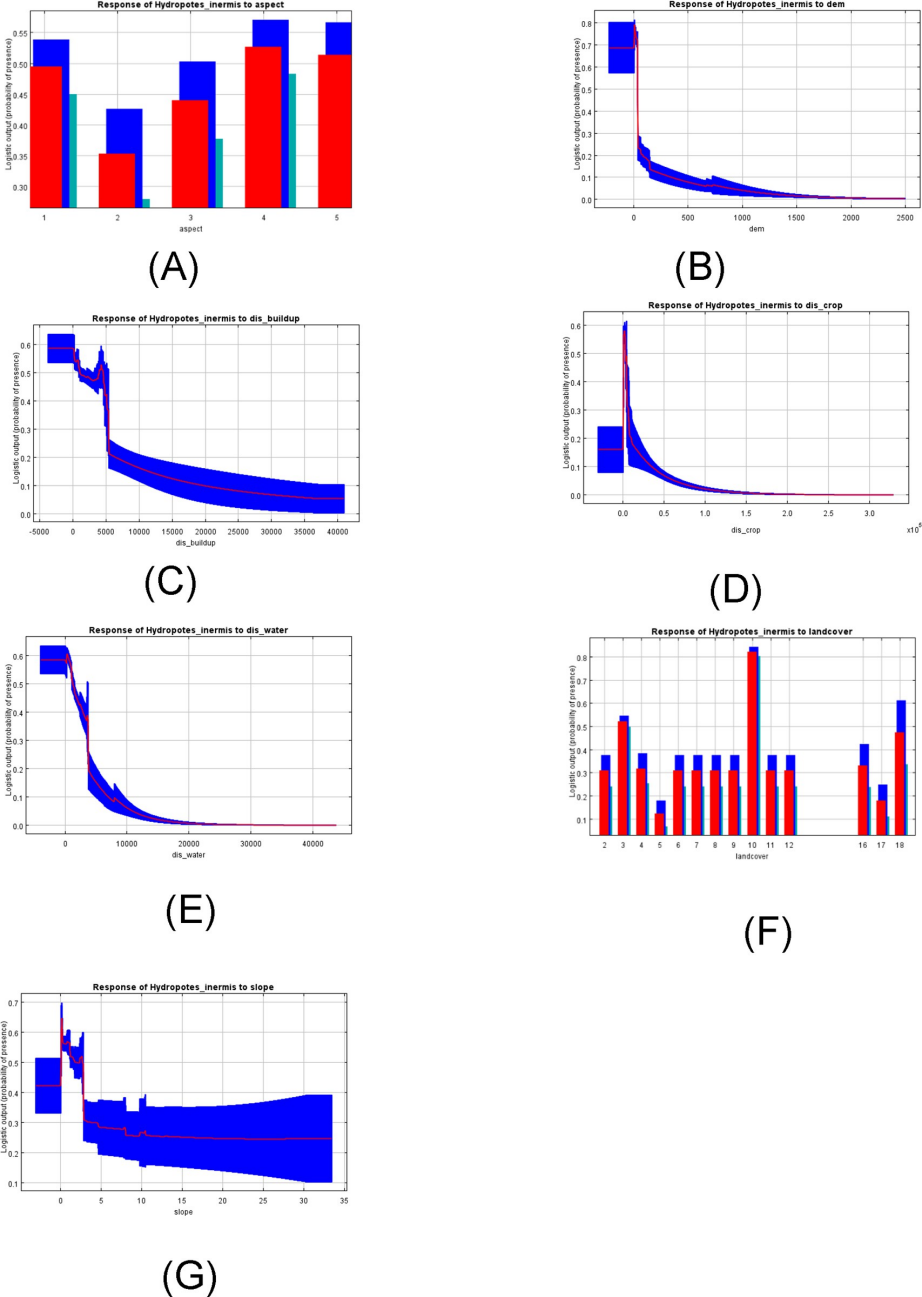
Response curves of water deer to environmental variables used for model prediction. (A: Aspect; B: DEM (Altitude); C: Distance to built-up; D: Distance to cropland; E: Distance to water; F: Land cover type; G:Slope).

Based on the response curves (Fig 2), from a logistic output value above 0.5, a higher probability of presence could be determined for each variable. Factors that contributed to the highest probability of water deer presence were elevation below 100 m, dense short vegetation, wetland with dense short vegetation and open tree cover, and distance below 1000 m from water source and croplands.

**Fig 3:**
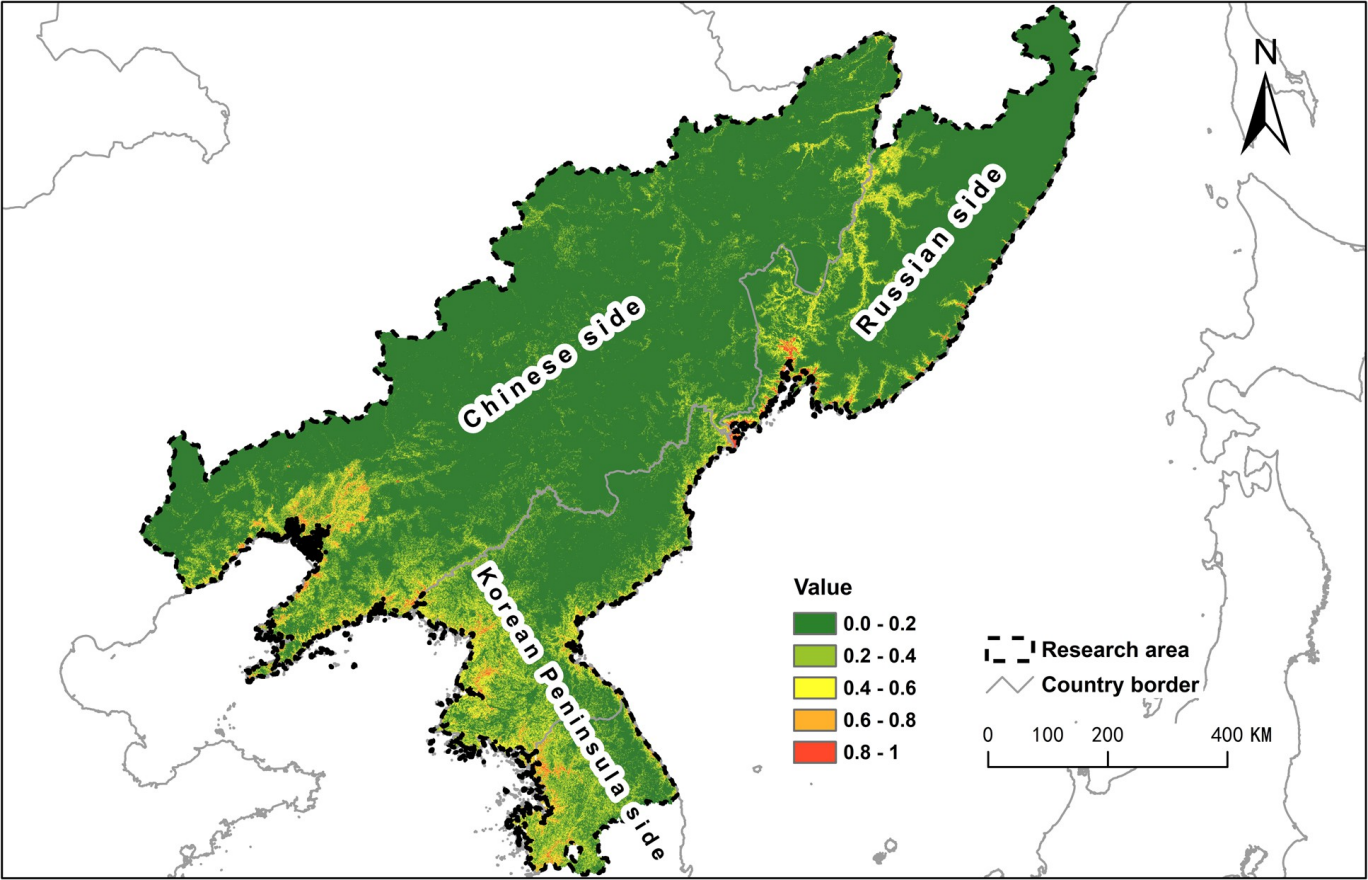
Maximum entropy model of habitat suitability for water deer (AUC = 0.939±0.011). The high suitability region (0.4–1) located along the Korean Peninsula’s east and west coasts towards northeast China and the Russian Far East. Map was computed using Maxent (version 3.4.; [44]) in ArcMap 10.3 (desktop.arcgis.com; ESRI, Redlands, USA) based on species data originating from author’s data (S1 Table) and published data source [12, 14, 34] as well as GBIF database (DOI: 10.15468/39omei). We used environmental layers from including landcover data from GLCLU (https://glad.umd.edu/dataset/global-land-cover-land-use-v1), water system data from OpenStreetMap (https://www.openstreetmap.org/), as well as topography data from SRTM (https://srtm.csi.cgiar.org/) and the detail data description was listed in Table 1.

The habitat with the highest suitability was mainly found in three areas (Fig 3). One comprised of the west coast of the Korean Peninsula, stretching into the southern Liaoning province in northeast China. The second was found along the east coast of the Korean Peninsula, ranging to the north of the Russian Far East coastal area. The third comprised the area in which water deer have recently distributed in the lower Tumen basin and stretched 740 km to the north along the Ussuri River. Some of the high-quality habitat was inside the protected area of China’s Northeast Tiger and Leopard National Park and the neighboring Land of Leopard National Park in Russia, at the same time, some suitable habitat downstream of the Tumen River was not included in any protected area.

### Habitat connectivity

**Fig 4:**
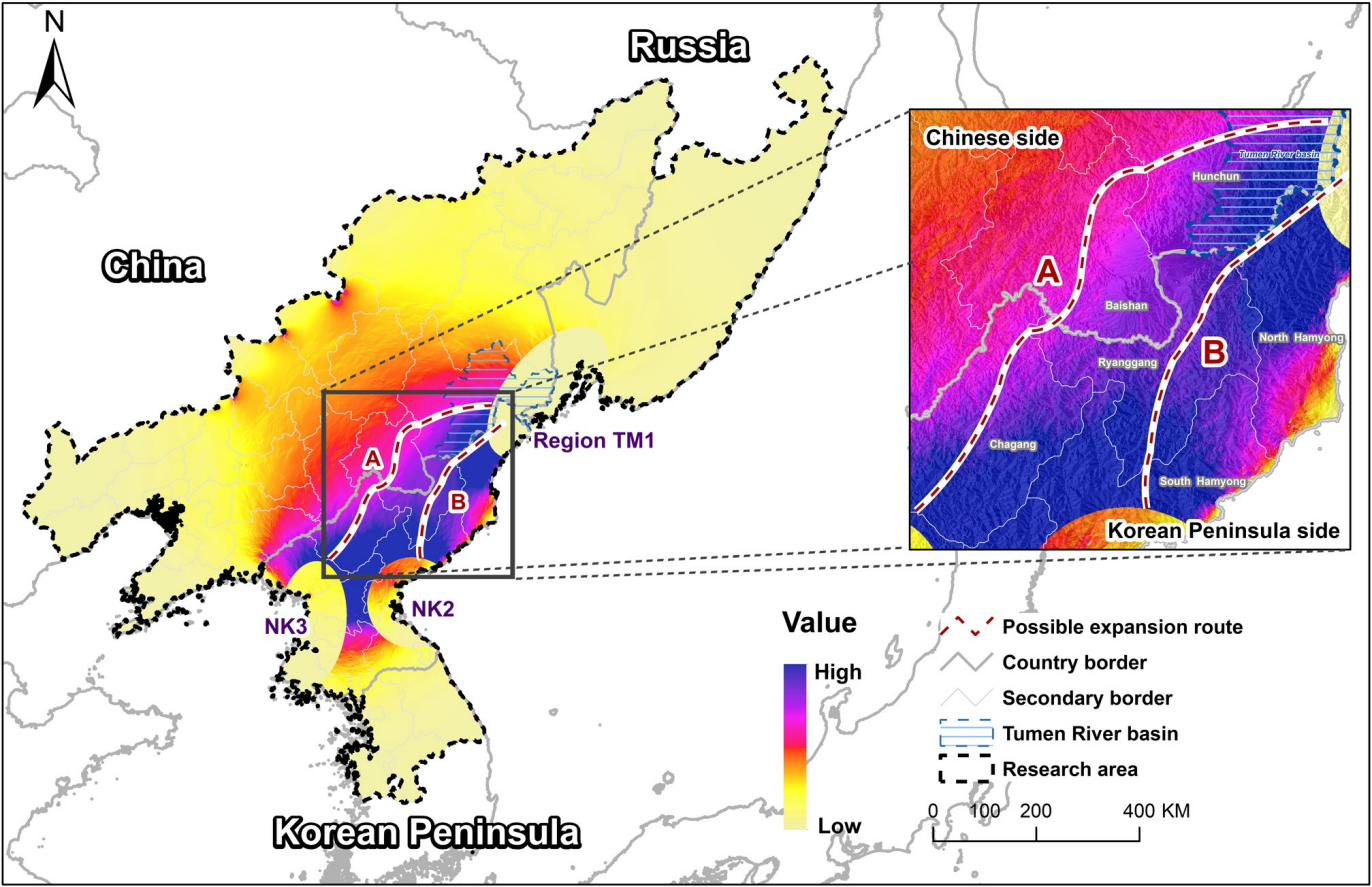
Possible dispersal routes of water deer. We calculated the connectivity between the suitable habitat patches for the regions in TM1, NK1 and NK2 and two possible routes, route A crosses North Korea and into China reaching the patch TM2, and route B crosses the east of North Korea and into the region TM1. Map was computed using Circuitscape ArcGIS toolkit [50] with the data from the MaxEnt result and mapped in ArcMap 10.3 (desktop.arcgis.com; ESRI, Redlands, USA).

Region TM1 comprised the areas newly occupied by water deer, located in the lower Tumen River basin. Region NK2 was in the western part of North Korea, including North Pyongan province, the western part of South Pyongan province, the Pyongyang region, and the western parts of North and South Hwanghae provinces. Region NK3 included the southern part of South Hamgyong province and the northern area of Kangwon province in eastern North Korea. Our results show strong connectivity between regions NK2 and NK3, but only weak connectivity between these two regions and region TM1. Two possible dispersion routes were estimated based on the landscape connectivity analysis. Route A possibly passes north of South Pyongan province and South Hamgyong province to Chagang province and passes the border between China and North Korean south of the Baishan area and through Linjiang, Fusong, and south of Antu, Wangqing, and Hunchun before reaching region TM1 downstream of the Tumen River region and connecting region NK2 to region NK3 (Fig 4). Route B connected the habitat between regions NK3 and TM1, whereas South Hamgyong Province, except the eastern area, connected to North Hamgyong province, through Ryanggang province and the border area between China and North Korea, and passes the northern area of North Hamgyong province to the north and connected to the region downstream of the Tumen River.

The route A crosses several protected areas in China: Baishan Musk Deer National Nature Reserve, the northern area of the Changbai mountain Nature Reserve; and Russia: The Tiger and Leopard National Park. For the sections of route A and B that cross North Korea, it is difficult to identify the precise route in or out of protected areas because no information on the boundaries of protected areas in North Korea is available.

## Discussion

### Suitable habitat

Water deer expansion into the transboundary region between the Russian Far East, northeast China, and North Korea is a recent event, and suitable habitat assessment provides insights into their expansion trends. Our study used current knowledge on this expansion combined with landscape factors to find suitable habitat in a larger area, including parts of the Korean Peninsula, northeast China, and the Russian Far East.

Our study predicted a suitable habitat in the east and west coasts of the Korean Peninsula, which was consistent with the information in the existing records. We analyzed the influence of landscape factors to find suitable habitats, which may explain the current expansion in the range of water deer. Based on our results, beyond the current occurrence area, suitable habitat extends to the north in the Russian Far East, where there is lower human pressure as well as good water resources and vegetation available. Water deer have high fecundity compared to other deer species. Each year, a female deer can give birth to an average of three to four calves, with a maximum of seven calves recorded, and the calves reach sexual maturity after one year [51]. With such a strong reproductive capacity, population numbers in the areas of expansion are likely to increase rapidly, and there is a high possibility that they will continue to expand their territory towards the north.

Low elevation, proximity to cropland and water, and presence of wetlands were important environmental factors according to our model. This may be related to physiology and diet of water deer as well as competition with other deer species such as roe deer, as body size and morpho-physiological characteristics are a direct factors of deer’s diet composition [52]. If a deer species is introduced to a new area, then the ecosystem will be influenced by the species and competition with local deer may be ensue [53, 54].

Our findings in context of land use variable result from habitat model match with the knowledge of the species as GPS tracking data from water deer research in South Korea showed that forest, wetland, agriculture, and water areas were the most frequently used land covers [55]. Further, the preference for 20–25° slopes and broadleaved forests was significantly related with water deer density [56]. Wetlands, water, and agricultural areas are landscape features of similar importance both in South Korea and the newly occupied region, although differences may arise due to varying geographical conditions in different areas. MaxEnt is suggested to be a highly accurate machine learning method to model habitat suitability for habitat prediction of water deer in South Korea [38]. In our research, MaxEnt performed well for predicting habitat and influencing factors.

### Dispersal routes

Based on the landscape connectivity analysis, two possible routes were estimated as having a high probability of water deer dispersal from the North Korean populations to the newly occupied region, and at the same time, no barriers could be observed between the two suitable areas in North Korea. The newly colonized region in the lower Tumen area was next to important protected areas, viz., the Northeast China Tiger and Leopard National Park, the Khasansky provincial nature park and the Land of the Leopard National Park in Russia. In North Korea, protected areas may also play an important role in the dispersal of wildlife, such as the Suryong Mountain Animal Reserve located in the Tosan County of North Hwanghae Province, which can connect the two suitable habitat patches region NK2 and region NK3. Along the predicted route B, protected areas such as Daeheung Animal Reserve and Donggye animal reserve are present in Ryanggang Province, and Gwanmobong nature reserve forest area in North Hamyeong province. Some protected areas for birds or fish may also contribute to the provision of suitable water resources and wetland habitat for water deer dispersal, such as Kuumya Migratory Bird Reserve in South Hamgyong Province, and Rason Migratory Bird Reserve in North Hamgeong Province, which connects the wetlands in the lower Tumen River with the wetlands in China and Russia. Water deer dispersion through the route A may cross central northern North Korea into China and reach the newly occupied area by moving along the border. Within North Korea, Geumseok animal reserve and Ogasan nature reserve may provide habitat for water deer dispersing northward across the Yalu River marking the China-North Korean border. From there, dispersing water deer can reach the forests in China in the Jilin Baishan Musk Deer National Nature Reserve or continue through the northern area of Changbai mountain to reach the Tiger and Leopard National Park, and subsequently reach the newly occupied region. The predicted route estimate is limited by the absence of data on animal movement, which could provide a better understanding of dispersal routes and habitat connectivity.

The water deer dispersal route may also provide insights for the conservation of big cats. The Changbai mountain region was predicted to be an important potential habitat for the recovery of an isolated Amur tiger population [57], and the connectivity between habitat patches is crucial for the success of their conservation. There is already evidence that Amur tigers and leopards have started hunting this new prey species in the newly colonized region. There are available evidences such as unpublished camera trap photographs of a leopard hunting water deer in China, and tiger scat with water deer teeth in Russia. Ecological research on tigers suggest the most important requirements for wild tiger survival are prey, water, and sheltered areas for hunting [58]. Prey availability is the most basic and important element for predators; thus, new prey habitats may contribute to the recovery of large cats in the landscape.

There are many barriers due to national security issues between countries in Northeast Asia, but the water deer expansion and our habitat connection analyses show that there is connectivity between sites for wildlife in the region. Future surveys may have important implications for the conservation of the connected landscape.

### Implications for conservation

Species based conservation in a single area is insufficient. Climate change and human activities drive species dispersal from one site to another. A connected landscape for wildlife movement and survival reduces the risk for species facing threats in a single region, as well as mitigates pressure from environmental influence. Conservation planning needs to move from a single-site approach to one that can incorporate processes at landscape level. In the case of water deer dispersal, a transboundary biodiversity conservation plan needs to be considered, as landscape connectivity in the whole region will contribute to the health of forest and wetland ecosystems.

Numerous issues can affect the success of landscape conservation, such as the lack of surveys, illegal hunting, unclear boundaries of reserves, lack of awareness among local people, and insufficient collaboration between stakeholders. Furthermore, there are many cases of water deer mortalities due to roadkill [59]; therefore, measures to prevent roadkill are also necessary. Based on the available information regarding water deer in South Korea, water deer consumes crops, rice, and other agricultural products, and in areas with high quality habitat, different measures are needed to prevent conflicts between water deer and the local community [51]. Other direct threats, such as illegal hunting, need to be addressed by raising local community awareness and through projects mitigating conflicts between wildlife and humans. Knowledge exchange between different stakeholders within and between countries will not only contribute to the better management of one species but will also benefit the whole landscape and the ecosystem. Our research explored the habitat suitability in the new range region, and landscape connectivity in the transboundary area. The results of our study can provide important information for future research, such as foraging behavior of water deer and landscape genetic-based study to examine the dispersal route functionality.

## Conclusion

In our research, we analyzed water deer data from newly occupied areas in the transboundary region between China, Russia, and North Korea, and predicted suitable habitats and two possible dispersal routes connecting them. With this information, we provide a general understanding of the habitat use of this species and demonstrate the existence of connectivity for wildlife in the larger landscape, which can contribute to the conservation of the whole landscape, although it will need more effort to survey and protect.

## Supporting information

S1 Table Water deer occurrence information used for the MaxEnt modelling. (DOCX)
