# Supplementary material for: Prediction of range expansion and estimation of dispersal routes of water deer (Hydropotes inermis) in the transboundary region between China, the Russian Far East and the Korean Peninsula
Source: PLoS One. 2022 Apr 14;17(4):e0264660. doi: 10.1371/journal.pone.0264660 (PMC9009690; doi:10.1371/journal.pone.0264660)
Supplement: S1 Table — (DOCX) [file pone.0264660.s001.docx]

**S1 Table. Water deer occurrence information used for the MaxEnt modelling.**

| **Speciese** | **Date** | **Date type** | **Description** | **Area** | **Longitude** | **Latitude** |
| --- | --- | --- | --- | --- | --- | --- |
| Hydropotes inermis | 2019-5-23 0:00 | Roadkill | Carcass | China | 130.2698498 | 42.87993325 |
| Hydropotes inermis | 2019-7-9 0:00 | Roadkill | Carcass | China | 130.4410297 | 42.55645642 |
| Hydropotes inermis | 2020-1-3 0:00 | Monitoring | Footprint,Urine | China | 130.0406492 | 43.51353691 |
| Hydropotes inermis | 2020-1-8 0:00 | Monitoring | Footprint,Urine | China | 130.5777533 | 42.65982599 |
| Hydropotes inermis | 2020-1-8 0:00 | Monitoring | Footprint,Urine | China | 130.5833805 | 42.65492392 |
| Hydropotes inermis | 2020-1-8 0:00 | Monitoring | Footprint,Urine | China | 130.5873661 | 42.65244712 |
| Hydropotes inermis | 2020-1-11 0:00 | Monitoring | Footprint,Urine | China | 130.5889578 | 42.64004759 |
| Hydropotes inermis | 2020-1-11 0:00 | Monitoring | Footprint,Urine | China | 130.6079687 | 42.61763426 |
| Hydropotes inermis | 2020-1-11 0:00 | Monitoring | Footprint,Urine | China | 130.6162708 | 42.60773811 |
| Hydropotes inermis | 2020-1-11 0:00 | Monitoring | Field observation | China | 130.5893737 | 42.63167561 |
| Hydropotes inermis | 2020-1-11 0:00 | Monitoring | Footprint,Urine | China | 130.5870366 | 42.63228229 |
| Hydropotes inermis | 2020-1-12 0:00 | Monitoring | Footprint,Urine | China | 130.4570337 | 42.60326126 |
| Hydropotes inermis | 2020-1-12 0:00 | Monitoring | Footprint,Urine | China | 130.4559985 | 42.58438668 |
| Hydropotes inermis | 2020-1-12 0:00 | Monitoring | Footprint,Urine | China | 130.4273548 | 42.57121093 |
| Hydropotes inermis | 2020-1-12 0:00 | Monitoring | Footprint,Urine | China | 130.4396724 | 42.6029194 |
| Hydropotes inermis | 2020-1-16 0:00 | Monitoring | Field observation | China | 129.9355245 | 43.01583867 |
| Hydropotes inermis | 2020-1-19 0:00 | Monitoring | Field observation | China | 130.3163226 | 42.94753019 |
| Hydropotes inermis | 2020-2-11 0:00 | Monitoring | Field observation | China | 130.4546412 | 42.59327572 |
| Hydropotes inermis | 2018 2019 | Camera trapping | Photo | China | 126.576325 | 41.62256944 |
| Hydropotes inermis | 2019 | Camera trapping | Photo | China | 126.4813667 | 41.7935 |
| Hydropotes inermis | 2019 | Camera trapping | Photo | China | 126.4712389 | 41.79670278 |
| Hydropotes inermis | 2019 | Camera trapping | Photo | China | 126.57395 | 41.62336111 |
| Hydropotes inermis | 2019 | Camera trapping | Photo | China | 126.5812889 | 41.62435556 |
| Hydropotes inermis | 2019 | Camera trapping | Photo | China | 126.5817611 | 41.62448333 |
| Hydropotes inermis | 2018.1.13 | Camera trapping | Photo | China | 126.5729253 | 41.62167314 |
| Hydropotes inermis | 2018.1.17 | Camera trapping | Photo | China | 126.5784867 | 41.62374407 |
| Hydropotes inermis | 2018.3.7 | Camera trapping | Photo | China | 126.5783997 | 41.62318407 |
| Hydropotes inermis | 2018.4.29 | Camera trapping | Photo | China | 126.5724488 | 41.62733477 |
| Hydropotes inermis | 2018.4.10 | Camera trapping | Photo | China | 126.5668854 | 41.62403913 |
| Hydropotes inermis | 2019 | Camera trapping | Photo | China | 126.4698288 | 41.79681615 |
| Hydropotes inermis | 2019 | Camera trapping | Photo | China | 126.4799521 | 41.79359989 |
| Hydropotes inermis | 2019 | Camera trapping | Photo | China | 126.4820795 | 41.79088283 |
| Hydropotes inermis | 2020-04-02; 04:06 | Camera trapping | photo | China | 130.58334 | 42.65495 |
| Hydropotes inermis | 2020-01-14; 14:24 | Camera trapping | photo | China | 130.60791 | 42.61762 |
| Hydropotes inermis | 2020-01-30; 16:15 | Camera trapping | photo | China | 130.58699 | 42.63227 |
| Hydropotes inermis | 2020-02-10; 11:36 | Camera trapping | photo | China | 130.58699 | 42.63227 |
| Hydropotes inermis | 2020-02-13; 07:18 | Camera trapping | photo | China | 130.58699 | 42.63227 |
| Hydropotes inermis | 2020-03-09; 10:38 | Camera trapping | photo | China | 130.58699 | 42.63227 |
| Hydropotes inermis | 2020-03-16; 23:53 | Camera trapping | photo | China | 130.58699 | 42.63227 |
| Hydropotes inermis | 2020-02-13; 11:51 | Camera trapping | photo | China | 130.45703 | 42.60326 |
| Hydropotes inermis | 2020-03-17; 07:32 | Camera trapping | photo | China | 130.43974 | 42.60297 |
| Hydropotes inermis | 2019-4-24 23:22 | Camera trapping | photo | China | 130.23895 | 42.98007 |
| Hydropotes inermis | 2020-10-28 3:26 | Camera trapping | photo | China | 130.14075 | 42.93641 |
| Hydropotes inermis | 2020-12-21 10:22 | Camera trapping | photo | China | 130.14075 | 42.93641 |
| Hydropotes inermis | 2019-5-27 23:14 | Camera trapping | photo | China | 130.18892 | 42.92532 |
| Hydropotes inermis | 2019-6-19 8:18 | Camera trapping | photo | China | 130.18892 | 42.92532 |
| Hydropotes inermis | 2021-2-4 21:06 | Camera trapping | photo | China | 130.21033 | 42.92532 |
| Hydropotes inermis | 2019-5-11 18:49 | Camera trapping | photo | China | 130.16322 | 42.92532 |
| Hydropotes inermis | 2019-5-20 4:53 | Camera trapping | photo | China | 130.16322 | 42.92532 |
| Hydropotes inermis | 2021-5-14 4:52 | Camera trapping | photo | China | 130.16322 | 42.92532 |
| Hydropotes inermis | 2021-5-14 6:53 | Camera trapping | photo | China | 130.21385 | 42.92532 |
| Hydropotes inermis | 2019-6-19 10:57 | Camera trapping | photo | China | 130.28911 | 42.92532 |
| Hydropotes inermis | 2020-12-22 22:39 | Camera trapping | photo | China | 130.28911 | 42.92532 |
| Hydropotes inermis | 2020-12-28 10:24 | Camera trapping | photo | China | 130.28911 | 42.92532 |
| Hydropotes inermis | 2020-4-23 17:13 | Camera trapping | photo | China | 130.20572 | 42.92532 |
| Hydropotes inermis | 2021-3-8 10:59 | Camera trapping | photo | China | 130.20572 | 42.92532 |
| Hydropotes inermis | 2021-4-30 12:18 | Camera trapping | photo | China | 130.20572 | 42.92532 |
| Hydropotes inermis | 2020-12-18 5:23 | Camera trapping | photo | China | 130.20691 | 42.92532 |
| Hydropotes inermis | 2021-5-31 8:29 | Camera trapping | photo | China | 130.20691 | 42.92532 |
| Hydropotes inermis | 2020-11-2 23:22 | Camera trapping | photo | China | 130.18655 | 42.92532 |
| Hydropotes inermis | 2021-6-5 21:04 | Camera trapping | photo | China | 130.18655 | 42.92532 |
| Hydropotes inermis | 2021-6-5 21:43 | Camera trapping | photo | China | 130.18655 | 42.92532 |
| Hydropotes inermis | 2021-6-27 19:53 | Camera trapping | photo | China | 130.18655 | 42.92532 |
| Hydropotes inermis | 2020-8-26 1:41 | Camera trapping | photo | China | 130.18221 | 42.92532 |
| Hydropotes inermis | 2021-6-24 4:46 | Camera trapping | photo | China | 130.14977 | 42.92532 |
| Hydropotes inermis | 2020-5-10 18:35 | Camera trapping | photo | China | 130.1245 | 42.92532 |
| Hydropotes inermis | 2020-5-23 10:30 | Camera trapping | photo | China | 130.1245 | 42.92532 |
| Hydropotes inermis | 2020-6-4 6:07 | Camera trapping | photo | China | 130.1245 | 42.92532 |
| Hydropotes inermis | 2020-6-6 4:07 | Camera trapping | photo | China | 130.1245 | 42.92532 |
| Hydropotes inermis | 2020-6-8 19:19 | Camera trapping | photo | China | 130.1245 | 42.92532 |
| Hydropotes inermis | 2020-6-23 20:13 | Camera trapping | photo | China | 130.1245 | 42.92532 |
| Hydropotes inermis | 2020-6-30 17:54 | Camera trapping | photo | China | 130.1245 | 42.92532 |
| Hydropotes inermis | 2020-7-12 3:05 | Camera trapping | photo | China | 130.1245 | 42.92532 |
| Hydropotes inermis | 2020-7-12 11:31 | Camera trapping | photo | China | 130.1245 | 42.92532 |
| Hydropotes inermis | 2020-7-13 15:09 | Camera trapping | photo | China | 130.1245 | 42.92532 |
| Hydropotes inermis | 2020-7-13 18:57 | Camera trapping | photo | China | 130.1245 | 42.92532 |
| Hydropotes inermis | 2020-7-23 5:24 | Camera trapping | photo | China | 130.1245 | 42.92532 |
| Hydropotes inermis | 2020-9-8 23:25 | Camera trapping | photo | China | 130.1245 | 42.92532 |
| Hydropotes inermis | 2020-9-9 8:52 | Camera trapping | photo | China | 130.1245 | 42.92532 |
| Hydropotes inermis | 2020-9-21 6:14 | Camera trapping | photo | China | 130.1245 | 42.92532 |
| Hydropotes inermis | 2020-10-15 4:37 | Camera trapping | photo | China | 130.1245 | 42.92532 |
| Hydropotes inermis | 2020-10-18 6:15 | Camera trapping | photo | China | 130.1245 | 42.92532 |
| Hydropotes inermis | 2020-10-18 17:55 | Camera trapping | photo | China | 130.1245 | 42.92532 |
| Hydropotes inermis | 2020-10-20 7:14 | Camera trapping | photo | China | 130.1245 | 42.92532 |
| Hydropotes inermis | 2020-10-23 5:32 | Camera trapping | photo | China | 130.1245 | 42.92532 |
| Hydropotes inermis | 2020-10-28 6:48 | Camera trapping | photo | China | 130.1245 | 42.92532 |
| Hydropotes inermis | 2020-11-4 9:15 | Camera trapping | photo | China | 130.1245 | 42.92532 |
| Hydropotes inermis | 2020-11-7 6:34 | Camera trapping | photo | China | 130.1245 | 42.92532 |
| Hydropotes inermis | 2020-11-7 15:58 | Camera trapping | photo | China | 130.1245 | 42.92532 |
| Hydropotes inermis | 2020-11-11 7:35 | Camera trapping | photo | China | 130.1245 | 42.92532 |
| Hydropotes inermis | 2020-11-25 7:40 | Camera trapping | photo | China | 130.1245 | 42.92532 |
| Hydropotes inermis | 2020-11-25 17:56 | Camera trapping | photo | China | 130.1245 | 42.92532 |
| Hydropotes inermis | 2020-12-4 16:34 | Camera trapping | photo | China | 130.1245 | 42.92532 |
| Hydropotes inermis | 2020-12-14 22:35 | Camera trapping | photo | China | 130.1245 | 42.92532 |
| Hydropotes inermis | 2020-12-17 13:52 | Camera trapping | photo | China | 130.1245 | 42.92532 |
| Hydropotes inermis | 2020-12-18 23:07 | Camera trapping | photo | China | 130.1245 | 42.92532 |
| Hydropotes inermis | 2020-12-22 9:34 | Camera trapping | photo | China | 130.1245 | 42.92532 |
| Hydropotes inermis | 2021-1-13 8:02 | Camera trapping | photo | China | 130.1245 | 42.92532 |
| Hydropotes inermis | 2021-2-25 13:49 | Camera trapping | photo | China | 130.1245 | 42.92532 |
| Hydropotes inermis | 2021-4-17 17:20 | Camera trapping | photo | China | 130.1245 | 42.92532 |
| Hydropotes inermis | 2021-4-26 16:13 | Camera trapping | photo | China | 130.1245 | 42.92532 |
| Hydropotes inermis | 2021-6-18 9:35 | Camera trapping | photo | China | 130.1245 | 42.92532 |
| Hydropotes inermis | 2020-11-23 8:47 | Camera trapping | photo | China | 130.12885 | 42.92532 |
| Hydropotes inermis | 2020-11-29 16:10 | Camera trapping | photo | China | 130.12885 | 42.92532 |
| Hydropotes inermis | 2020-12-19 13:50 | Camera trapping | photo | China | 130.12885 | 42.92532 |
| Hydropotes inermis | 2020-12-29 7:25 | Camera trapping | photo | China | 130.12885 | 42.92532 |
| Hydropotes inermis | 2021-1-17 10:38 | Camera trapping | photo | China | 130.12885 | 42.92532 |
| Hydropotes inermis | 2018-3-21 7:29 | Camera trapping | photo | China | 130.15228 | 42.92532 |
| Hydropotes inermis | 2019 11 06 | monitoring | visual observation | Russia | 130.56824 | 42.72826 |
| Hydropotes inermis | 2019 08 09 | monitoring | visual observation | Russia | 130.58044 | 42.70203 |
| Hydropotes inermis | 2019 12 13 | monitoring | visual observation | Russia | 130.60253 | 42.51696 |
| Hydropotes inermis | 2019 12 13 | monitoring | from helicopter | Russia | 130.60498 | 42.51165 |
| Hydropotes inermis | 2019 10 04 | monitoring | photograph | Russia | 130.60562 | 42.67994 |
| Hydropotes inermis | 2019 10 29 | monitoring | photo | Russia | 130.60838 | 42.64196 |
| Hydropotes inermis | 2019 12 13 | monitoring | from helicopter | Russia | 130.61074 | 42.50276 |
| Hydropotes inermis | 2019 12 13 | monitoring | from helicopter | Russia | 130.61138 | 42.49987 |
| Hydropotes inermis | 2018 09 10 | monitoring | visual observation | Russia | 130.61216 | 42.68181 |
| Hydropotes inermis | 2019 03 10 | monitoring | visual observation | Russia | 130.62113 | 42.49879 |
| Hydropotes inermis | 2019 11 07 | monitoring | visual observation | Russia | 130.63203 | 42.63052 |
| Hydropotes inermis | 2019 07 11 | monitoring | visual observation | Russia | 130.63582 | 42.43372 |
| Hydropotes inermis | 2019 10 06 | monitoring | visual observation | Russia | 130.65116 | 42.64627 |
| Hydropotes inermis | 2019 11 11 | monitoring | visual observation | Russia | 130.65521 | 42.4318 |
| Hydropotes inermis | 2019 11 07 | monitoring | visual observation | Russia | 130.66235 | 42.40067 |
| Hydropotes inermis | 2019 11 07 | monitoring | visual observation | Russia | 130.66373 | 42.32211 |
| Hydropotes inermis | 2019 11 07 | monitoring | photo | Russia | 130.66573 | 42.31804 |
| Hydropotes inermis | 2019 11 23 | monitoring | visual observation | Russia | 130.67722 | 42.4525 |
| Hydropotes inermis | 2019 11 08 | monitoring | visual observation | Russia | 130.68763 | 42.55082 |
| Hydropotes inermis | 2019 10 05 | monitoring | visual observation | Russia | 130.69897 | 42.34077 |
| Hydropotes inermis | 2019 11 11 | monitoring | visual observation | Russia | 130.69975 | 42.57195 |
| Hydropotes inermis | 2019 11 05 | monitoring | visual observation | Russia | 130.70002 | 42.69388 |
| Hydropotes inermis | 2019 09 18 | monitoring | visual observation | Russia | 130.70111 | 42.58667 |
| Hydropotes inermis | 2019 11 08 | monitoring | visual observation | Russia | 130.7017 | 42.56922 |
| Hydropotes inermis | 2019 05 23 | monitoring | visual observation | Russia | 130.7048 | 42.34822 |
| Hydropotes inermis | 2019 08 28 | monitoring | visual observation | Russia | 130.71092 | 42.38357 |
| Hydropotes inermis | 2019 10 05 | monitoring | visual observation | Russia | 130.7149 | 42.34165 |
| Hydropotes inermis | 2019 12 13 | monitoring | from helicopter | Russia | 130.7153 | 42.57071 |
| Hydropotes inermis | 2019 05 05 | monitoring | visual observation | Russia | 130.71656 | 42.3491 |
| Hydropotes inermis | 2019 11 19 | monitoring | deregistration | Russia | 130.73781 | 42.3481 |
| Hydropotes inermis | 2019 04 05 | monitoring | visual observation | Russia | 130.75025 | 42.71531 |
| Hydropotes inermis | 2019 11 02 | monitoring | visual observation | Russia | 130.77656 | 42.72316 |
| Hydropotes inermis | 2019 12 13 | monitoring | from helicopter | Russia | 130.79283 | 42.54652 |
| Hydropotes inermis | 2019 10 20 | monitoring | visual observation | Russia | 130.83307 | 42.80312 |
| Hydropotes inermis | 2019 11 29 | monitoring | photo | Russia | 131.03647 | 42.71889 |
| Hydropotes inermis | 2019 12 25 | monitoring | visual observation | Russia | 131.30331 | 42.84975 |
| Hydropotes inermis |  | Monitoring |  | Russia | 130.79283 | 42.54652 |
| Hydropotes inermis |  | Camera trapping | photo | Russia | 130.60562 | 42.67994 |
| Hydropotes inermis |  | Camera trapping | photo | Russia | 130.60838 | 42.64196 |
| Hydropotes inermis |  | Camera trapping | photo | Russia | 130.60115 | 42.51112 |
| Hydropotes inermis |  | Camera trapping | photo | Russia | 130.59747 | 42.50564 |
| Hydropotes inermis |  | Monitoring |  | Russia | 130.60626 | 42.63669 |
| Hydropotes inermis | 2019 | Monitoring |  | Russia | 130.93498 | 42.826 |
| Hydropotes inermis | 2019-12-11 | Hunting information |  | Russia | 130.47771 | 42.33729 |
| Hydropotes inermis | 2019-12-25 | Hunting information | Hunted as roe deer | Russia | 130.43504 | 42.34924 |
| Hydropotes inermis | 2020-2-15 | Hunting information | Confiscated from poaching | Russia | 13065602 | 42.41273 |
